# Inference of spatiotemporal effects on cellular state transitions from time-lapse microscopy

**DOI:** 10.1186/s12918-015-0208-5

**Published:** 2015-09-21

**Authors:** Michael K. Strasser, Justin Feigelman, Fabian J. Theis, Carsten Marr

**Affiliations:** Institute of Computational Biology, Helmholtz Zentrum München, German Research Center for Environmental Health, Ingolstädter Landstr. 1, Neuherberg, 85764 Germany; Department of Mathematics, Technische Universität München, Boltzmannstr. 3, Garching, 85747 Germany

**Keywords:** Cell state transition, Time-lapse microscopy, Single cell analysis, LASSO, Spatial interaction

## Abstract

**Background:**

Time-lapse microscopy allows to monitor cell state transitions in a spatiotemporal context. Combined with single cell tracking and appropriate cell state markers, transition events can be observed within the genealogical relationship of a proliferating population. However, to infer the correlations between the spatiotemporal context and cell state transitions, statistical analysis with an appropriately large number of samples is required.

**Results:**

Here, we present a method to infer spatiotemporal features predictive of the state transition events observed in time-lapse microscopy data. We first formulate a generative model, simulate different scenarios, such as time-dependent or local cell density-dependent transitions, and illustrate how to estimate univariate transition rates. Second, we formulate the problem in a machine-learning language using regularized linear models. This allows for a multivariate analysis and to disentangle indirect dependencies via feature selection. We find that our method can accurately recover the relevant features and reconstruct the underlying interaction kernels if a critical number of samples is available. Finally, we explicitly use the tree structure of the data to validate if the estimated model is sufficient to explain correlated transition events of sister cells.

**Conclusions:**

Using synthetic cellular genealogies, we prove that our method is able to correctly identify features predictive of state transitions and we moreover validate the chosen model. Our approach allows to estimate the number of cellular genealogies required for the proposed spatiotemporal statistical analysis, and we thus provide an important tool for the experimental design of challenging single cell time-lapse microscopy assays.

**Electronic supplementary material:**

The online version of this article (doi:10.1186/s12918-015-0208-5) contains supplementary material, which is available to authorized users.

## Background

Cellular plasticity is the key property essential for multi-cellular development [1], tissue maintenance [2] and regeneration [3]. While the notion of state transitions from multipotent stem cells to mature functional cells is established, the breakthrough findings on transdifferentiation [4] and reprogramming [5] have sparked renewed interest into mechanisms driving cellular lineage choice with the prospect of therapeutic application [6].

To understand differentiation kinetics and thus the origins of stem cell population heterogeneity, one has to observe the transition of cells between states of different lineage potential. However, observing cell state transitions is impossible in data obtained from a single time point, emerging from e.g. flow cytometry, transcriptome or immunofluorescence analyses. For example, a clonal colony of differentiated cells may have originated from a single differentiated cell following multiple divisions, or from the simultaneous differentiation of multiple cells after a few divisions. Continuous time information and the tracking of individual cells is necessary to distinguish the two possibilities.

**Fig. 1:**
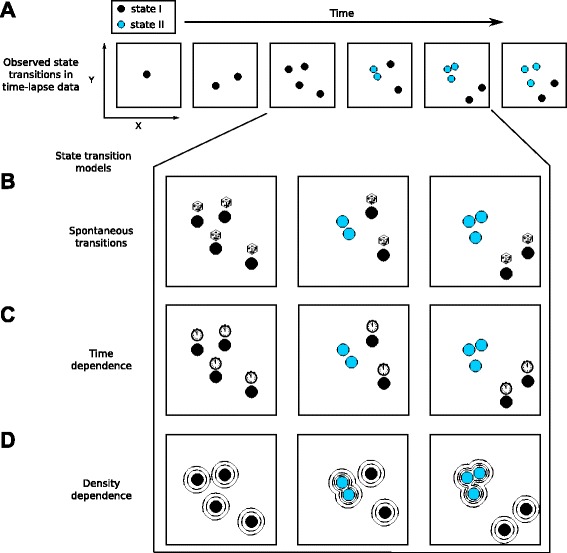
State transitions observed via time-lapse microscopy can be explained by different mechanisms. **a** During a time-lapse microscopy experiment cells are imaged over multiple time points. From these images, spatial configuration, cell proliferation and changes in cell state, e.g. via surface markers (we consider only two states, indicated by black and cyan) can be obtained. However, these observations do not inform about the underlying mechanisms that caused the transition in cell state. For example, the state transition could be entirely random (**b**), where cells spontaneously undergo state transitions (indicated by dice), it could depend on time (**c**), such that the transition rate changes in the course of the experiment (indicated by clocks). Alternatively, the transition could depend on local cell density (**d**), e.g. cells with higher local cell density preferentially transit from one state to the other

Live cell imaging allows to observe state transitions e.g. via cell surface markers or cell morphology (Fig. 1a), but it cannot immediately provide a mechanistic explanation why the transition occurs. For example, the differentiation rate of a stem cell towards a more mature cell type may depend on time [7] (Fig. 1c), cell density [8] (Fig. 1d), the makeup of surrounding niche cells [9] or on a combination of these features. While it is possible to quantify the emergence of cellular patterns in colonies [10, 11], it is impossible to tell from the mere observation if the simultaneous differentiation of multiple cells is a random event or if it is triggered by, e.g., the increased density in the colony. The inference of features predictive of this state transition rate requires robust statistical analysis, and thus a large number of time-lapse microscopy data, which is in particular for mammalian systems still a challenging and labor intensive task [12, 13].

Here, we present a model and analysis framework that can infer the spatiotemporal features predictive of state transitions and also allows to estimate the number of samples required for this analysis. To validate the performance of our framework, we first simulate cellular genealogies from a generative spatiotemporal model for different scenarios of transition rate dependencies. We then develop an inference method based on generalized linear models (GLM) and feature selection with *L*_1_ regularization. We show that our method is able to correctly identify the transition rate as a multi-feature function and determine the number of required genealogies and allowed tracking errors for different scenarios. Finally, we use the correlations between cell siblings to validate the chosen approach and detect shortcomings – either due to non-considered features, or due to cell-internal effects that drive cell state transitions.

## Methods

### A generative model for spatiotemporal cellular genealogies

**Fig. 2:**
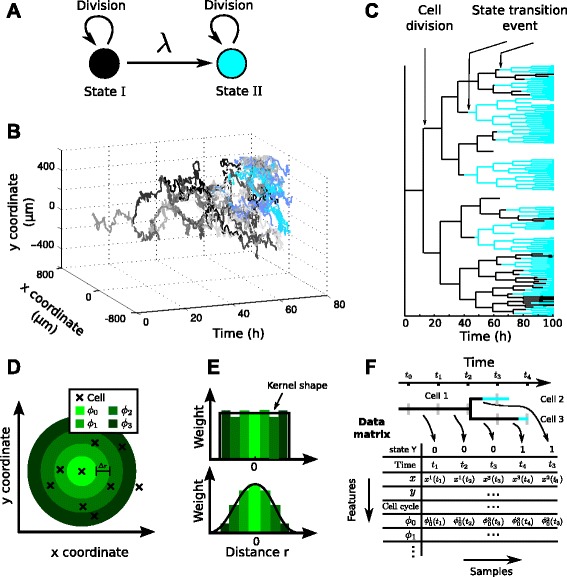
Spatiotemporal simulation and analysis of cell state transitions. **a** In our model, a cell in state I (black) can divide or transition into state II (cyan). The transition is governed by the transition rate *λ*, which can depend on features like time, position, cell cycle, or the local cell density. This unidirectional transition model is inspired by cellular differentiation where a undifferentiated progenitor cell irreversibly transitions into a more differentiated cell type. **b** Visualization of a cellular genealogy in space and time with cells in state I (black to gray) and state II (cyan to blue). **c** Tree view of the genealogy depicted in **b** (coloring as in **a**). **d** Local cell density is modeled via a set of annular basis functions *ϕ*_*k*_ with inner radii *k* *Δ* *r* and constant thickness *Δ* *r* (green circles). Cells are indicated as crosses. **e** Linear combinations of the *ϕ*_*k*_ can approximate any density dependence (e.g. a tophat kernel, upper panel, or a Gaussian kernel, lower panel). **f** The tree structured data is transformed into a data matrix by discretizing time (*t*_0_,…,*t*_4_ in this example) and creating one sample (i.e. one column) for each cell at each time interval, simulating a measurement process. For each cell *i* and each timepoint *t*, we record different features (i.e. rows), e.g. cell coordinates *x* ^*i*^(*t*) and *y* ^*i*^(*t*), the spatial features ${\phi ^{i}_{0}}(t), {\phi ^{i}_{1}}(t),\ldots $ (illustrated in **d**) and state transition events *Y* within the time interval

Throughout this paper, we use a simple model of cell state transition with two cellular states I and II (Fig. 2a). A single cell in state I (black circle in Fig. 2a) can divide into two cells in state I, or transition into another state II (cyan circle), where it can only divide. This unidirectional state transition could for example model cell differentiation, where a progenitor transforms into a more differentiated cell type, but the reverse transition does not occur naturally. The transition rate *λ*(*t*,*F*_*i*_(*t*)) of a cell *i* from state I to state II depends on the features *F*_*i*_ of the cell. Notably, the features *F*, like time, cell cycle state, position or local cell density, can change over time. Specific examples of the function *λ*(*t*,*F*_*i*_(*t*)) are introduced later on (see section “Cell state transition scenarios”).

Mathematically, in our model a single cell is defined by its 2D spatial coordinates $x \in \mathbb {R}^{2}$ (assuming an in vitro experimental setting where cells are imaged on a cover-slip), its state *Y*∈ [0,1], where *Y*=0 (*Y*=1) if the cell is in state I (II) and its age $\tau \in \mathbb {R}^{+}$, i.e. the time since the last division. The cell division rate *γ*(*τ*) is age dependent to account for non-exponential lifetime of cells (constant *γ* would yield unrealistic exponential lifetimes). This system of dividing cells that undergo state transitions evolves probabilistically in time and has to be described by a Master Equation (accounting not only for changes in *Y* and *x* but also considering cell divisions), whose derivation is sketched in Additional file 1: Section 1. Instead of solving the intractable Master Equation, we simulated realizations of the underlying stochastic process (Fig. 2b): Since the system has continuous (space *x*, age *τ*) and discrete (cell state *Y*) variables, a standard stochastic simulation algorithm cannot be applied and a hybrid simulation method must be used (see e.g. Haseltine et al. [14]). Cell position is treated as Brownian motion (movement speed resembles agile cells, e.g. hematopoietic stem and progenitor cells) and is updated via an Euler-Maruyama scheme [15].

To evolve the cell state in time for a single cell in state I, the simulation proceeds in small time steps *Δt*, during which a state transition event takes place with probability (see Additional file 1: Section 5)

$$\begin{array}{*{20}l} P_{i}(t)&=1-e^{-\int_{t}^{t + \Delta t} \lambda(t', F_{i}(t')) dt'} \approx 1-e^{-\lambda(t, F_{i}(t))\cdot \Delta t} \end{array} $$

for some arbitrary, state and time-dependent transition rate *λ*(*t*,*F*_*i*_(*t*)). The rate *λ* is evaluated at the beginning of each iteration, and the time step *Δt* is chosen sufficiently small (such that no appreciable change in cell locations occurs and the rate *λ* is approximately constant). The cell divides after 12 hours on average, corresponding to the typical lifetime of mammalian stem and progenitor cells [16, 17] (for simplicity, but without loss of generality, we assumed cell lifetime to be uniformly distributed in the interval [10 *h*,14 *h*]). The cell division replaces the dividing cell by two daughter cells, with positions close to that of the mother cell and with the same cell state: e.g. a mother cell in state I gives rise to two daughters in state I. These cells are then simulated in parallel. Over the course of the simulation, a cellular genealogy with a distinct cell state pattern emerges (Fig. 2c). Genealogies are simulated for 100 hours (8−9 generations of cells) corresponding to the typical observation periods of long term time-lapse microscopy [17–19].

### Local cell density

Local cell density for a single cell is estimated using a kernel *f* that determines how much each cell contributes to the local density at a certain point *x* in space as a function of intercellular distance. We define the local cell density ${\rho _{i}^{f}}(t)$ of cell *i* at time *t* with respect to a kernel $f:\mathbb {R}\rightarrow [0,\infty ]$:

(1) $$\begin{array}{*{20}l} {\rho_{i}^{f}}(t) = \sum_{j\ne i} f\left[ d(x_{i}(t),x_{j}(t)) \right]\;, \end{array} $$

where *x*_*i*_(*t*) is the spatial coordinate of cell *i* at time *t* and *d*(*x*_*i*_,*x*_*j*_) denotes Euclidean distance. We use either a tophat kernel (Fig. 2e, upper panel, black line) with

(2) $$ f(r) = I(r<R)\;,   $$

where *I*(…) is the indicator function of [0,1], or a Gaussian kernel (Fig. 2e, lower panel, black line) with

(3) $$ f(r) = \frac{1}{\sqrt{2\pi}\sigma}e^{-\frac{r^{2}}{2 \sigma^{2}}}\;.   $$

For the tophat kernel each cell within distance *R* contributes equally to the local density experienced by cell *i*, whereas cells with distance larger than *R* do not contribute at all. For the Gaussian kernel the contribution to the local cell density decreases smoothly with distance.

### Local cell density as a linear combination of basis functions

In order to model and estimate any (radially symmetric) density kernel *f*, we approximate *f* as a linear combination of basis functions *ϕ*_*k*_, *k*=0,1,…

(4) $$\begin{array}{*{20}l} f \approx \sum_{k} \omega_{k} \cdot \phi_{k}\;,  \end{array} $$

where the *ϕ*_*k*_ are defined as

$$\begin{array}{*{20}l} \phi_{k}(r) = I\left[ k \Delta r < r \le (k+1)\Delta r \right]\;, \end{array} $$

and *I*(…) denotes the indicator function. *ϕ*_*k*_ resembles a ring of inner radius *kΔr* and thickness *Δr* (Fig. 2d). For example, we can recover the tophat kernel with radius *R* (Eq. 2) by choosing the coefficients *ω*_*k*_ as

$$\begin{array}{*{20}l} \omega_{k} = \begin{cases} 1,& k\Delta r< R\\ 0,& k\Delta r\ge R \end{cases}\;. \end{array} $$

For our analysis, we choose *Δr*=40 *μm*, which allows to resolve short range interactions on the order of eukaryotic cell diameter (≈20 *μm*) but also long range interactions due to diffusive signaling molecules (max. 25 cell diameters or 500 *μm* [20]).

### Cell state transition scenarios

We create four datasets corresponding to different scenarios of cell state transition:

1. We consider a scenario where the transition rate is constant (*λ* constant), resembling spontaneous transitions independent of other effects:

(5) $$ \lambda(t,F_{i}(t)) = c\;,   $$

with *c*=0.01 *h*^−1^. Thus, a state transition in a cell with a typical 12 *h* lifetime will occur with probability *p*=0.11.

2. For a time-dependent scenario (*λ*∝ time), the transition rate is chosen as

(6) $$ \lambda(t,F_{i}(t)) = a \cdot t\;,  $$

i.e. linearly increasing with time (*a*=3·10^−4^*h*^−2^). Note that *λ* does not depend on any other feature *F* of the cell. A time-dependent transition rate might for example be encountered in an in vitro stem cell system, where primary stem cells are isolated, separated from the stem cell niche. Over time the stem cells are depleted of crucial signaling molecules previously supplied by the niche cells and start transitioning into more mature cells.

3. For a density-dependent scenario (*λ*∝ density), the local density of a cell *i* at time *t* is mediated by a tophat kernel (Eq. 2) with *R*=300 *μm*, which is roughly the distance a cell can move in its lifetime (we assume agile, non-adherent cells in our simulations). The transition rate *λ* is then defined by

(7) $$ \lambda\left(t, \rho_{i}^{\text{tophat}}(t)\right) = b \cdot \rho^{\text{tophat}}_{i}(t)\;,   $$

with *b*=0.002 *h*^−1^. Density-dependent transition rates might be relevant for in vitro cultures of embryonic stem cells, which are known to differentiate when cell density becomes too large [8]. Another example for density-dependent transitions are bacteria that use quorum sensing to estimate local cell density and base their fate decision on that, e.g. by becoming virulent [21].

4. For a time and density-dependent scenario (*λ*∝ density + time), the contributions of the previous two factors are summed, using a Gaussian kernel (Eq. 3 with *σ*=130 *μm*) to define cell density:

(8) $$ \lambda\left(t, \rho^{\text{Gauss}}_{i}(t)\right) = a \cdot t + b \cdot \rho^{\text{Gauss}}_{i}(t)\;.   $$

### Non-parametric estimation of the transition rate

Given a dataset as described above, we now delineate two methods to estimate the transition rate from the data. First, the transition rate *λ* can be estimated non-parametrically by considering the definition of the rate as the probability of a transition in an infinitesimal time *dt*:

(9) $$ P(t,t+dt| F_{i}(t)) = \lambda(t,F_{i}(t)) \cdot dt\;,   $$

where *P*(*t*,*t*+*dt*|*F*_*i*_(*t*)) is the probability for a transition in the interval [*t*,*t*+*dt*] in the presence of the features *F*. We estimate the probability *P*(*t*,*t*+*dt*|*F*) of a state transition in [*t*,*t*+*dt*] given features *F* as

$$\begin{array}{*{20}l} \hat P(t,t+dt| F) = \frac{\text{Number of transition events}| (t,F)}{\text{Number of cells in state I}| (t,F)}\;, \end{array} $$

which is the fraction of candidate cells (in state I) that transit into state II in [*t*,*t*+*dt*] having features *F*. After rearranging Eq. 9, we obtain

(10) $$\begin{array}{*{20}l} \hat \lambda(t,F)=\frac{1}{\Delta t} \cdot \frac{\text{Number of transition events}| (t,F)}{\text{Number of cells in state I}| (t,F)}  \end{array} $$

To measure the uncertainty of the estimates, we calculate Bayesian credibility intervals (see Additional file 1: Section 2).

### Estimating the transition rate via generalized linear models

The transition rate can be inferred systematically using a machine-learning framework. We consider every timepoint of each cell as an observed sample (*F*^(*i*)^,*Y*^(*i*)^), where *F*^(*i*)^ is a set of features measured for this sample (absolute time, time since last division, absolute spatial coordinates, and different measures of local cell density *ϕ*_*k*_). We use superscripts to index the samples to clearly distinguish it from the per-cell indexing via subscripts used previously. *Y*^(*i*)^∈{0,1} denotes the class label of the sample being either “state I” (*Y*^(*i*)^=0) or “transition into II” (*Y*^(*i*)^=1). A sample is considered as *Y*^(*i*)^=1 if a state transition occurred in the time interval of the sample. Timepoints after the state transition (either of the cell itself or its progeny) are discarded (Fig. 2f) since we are interested in what actually triggers the transition of cells, not the state of the cell itself. Counter-intuitively, all samples (*F*^(*i*)^,*Y*^(*i*)^) are independent, even though, e.g. adjacent samples typically are strongly correlated with respect to their features (Additional file 1: Section 3).

We use generalized linear models (GLMs, [22]) to learn the relation between features *F*^(*i*)^ and class labels *Y*^(*i*)^ as

$$\begin{array}{*{20}l} \mathbb{E}(Y^{(i)}|F^{(i)},w) = \mu^{(i)} = g^{-1}(w^{T}F^{(i)})\;, \end{array} $$

where *μ*^(*i*)^ is the expected value of an exponential family distribution, *g*^−1^ is called the mean function, and *w* is the weights vector that has to be learned from the data. Choosing a Bernoulli distribution and an exponential mean function would exactly correspond to our data generating process (Additional file 1: Section 4). However, this specific GLM has unfavorable numerical properties leading to convergence issues [23]. Therefore, we resort to a GLM that has the desired exponential mean function but a Poisson instead of a Bernoulli distribution (also known as Poisson regression) and has better numerical properties. Note that Poisson regression is generally used to model count data (where $Y^{(i)} \in \mathbb {N}_{0}$), but is a good approximation to binary data (*Y*^(*i*)^∈{0,1}) in the case of rare events (see Additional file 1: Section 4). Thus, we obtain the following log-likelihood (see Additional file 1: Section 4 for a derivation):

(11) $$\begin{array}{*{20}l}{} \log p(Y|F,w)=\sum_{i} \left[Y^{(i)} w^{T} F^{(i)} -e^{w^{T} F^{(i)}}-\log(Y^{(i)}!)\right]\;. \end{array} $$

### Feature selection via *L*_1_ regularization

**Fig. 3:**
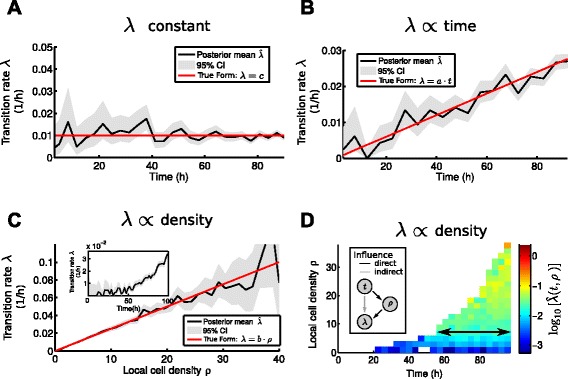
Features regulating the transition rate *λ* can be estimated non-parametrically from cellular genealogies with annotated state transition events. **a** The transition rate estimated from 100 genealogies (posterior mean, black line) agrees well with the true constant transition rate (red line). Gray areas indicate the 95 % credibility region of the estimate. **b** The transition rate estimated from 100 genealogies simulated with linear time-dependent rate agrees well with the true rate (red solid line). **c** The transition rate as a function of local cell density *ρ* for 100 genealogies simulated with density-dependent rate. The estimated transition rate seems to depend on both local density *ρ* (in line with the simulated form *λ*=*b*·*ρ*) and time (see inset). **d** The estimated transition rate $\hat \lambda $ as a function of both density and time reveals that the time-dependence observed in the inset in **c** is an indirect influence (density increases with time, see inset). Instead, the transition rate depends only on local cell density *ρ* (as seen by the predominantly uniform pattern of $\hat \lambda $ in time for fixed *ρ*, indicated by arrow)

**Fig. 4:**
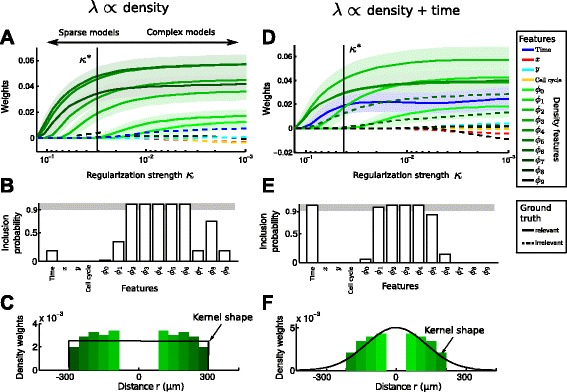
Regularized generalized linear models (GLMs) select the relevant features predictive of cell state transitions. **a** Regularization path of the GLMs applied to the density dependent dataset. The means (lines) and standard deviations (shaded regions, shown only for the relevant features) of the regression weights *w* are plotted against the regularization strength *κ* across 50 bootstrap samples (see Methods for details). The mean of the optimal regularization strength *κ* ^∗^ determined by cross validation is shown as a vertical black line. Solid (dashed) lines correspond to relevant (irrelevant) features in the respective scenario. **b** Percentage of bootstrap samples that included the respective features. Included features were determined as those with non zero weights at *κ* ^∗^. Enforcing a 90 % threshold (gray area) on the inclusion probability for each feature, we select the relevant features of the model. The features *ϕ*_0_,*ϕ*_1_ are not included as their effect is too weak to be detected by the GLM at the current sample size (see main text). **c** Reconstructed kernel of local cell density (bars) from the selected features in **b**. The true underlying tophat kernel shape is shown in black. As in **b**, the features *ϕ*_0_,*ϕ*_1_ are not included because their effect is to weak. **d**-**f** Analogous to **a**-**c**, but for a dataset where the transition rate *λ* depends on time and local cell density with a Gaussian kernel. Both features are correctly identified and the density kernel is correctly estimated

To determine the relevant features of the transition rate and to exclude features that only indirectly influence the state transition (as e.g. for scenario 3 with a density dependent *λ*, where however *λ* also indirectly depends on time; see Fig. 3c, d and Results), we apply *L*_1_ regularization to the GLM, also known as Lasso (least absolute shrinkage and selection operator) [24]. Here one minimizes the following function with respect to the weights *w*:

(12) $$\begin{array}{*{20}l} g(w) &= -\log p(Y|F,w) + \kappa \cdot \|w\|_{1}\;, \end{array} $$

with $\|w\|_{1} = \sum _{i} |w_{i}|$. This regularization is equivalent to placing a Laplace prior with location parameter *m*=0 and scale parameter *b*=*κ*^−1^ on the weights [25], resembling our knowledge that most of the weights should be zero and the resulting model should be sparse. Depending on the chosen regularization strength *κ*, one obtains models of differing sparsity (Fig. 4a). We follow the standard approach to determine the optimal regularization parameter *κ*^∗^: for each *κ*, we perform ten-fold cross validation using the deviance of the model as the error criterion and choose *κ*^∗^ based on the 1SE rule [26]: We select the largest *κ* (hence the simplest model) that in terms of its deviance is still within one standard error of the best *κ*. Optimization and cross validation of Lasso is performed using the function lassoglm() from the Matlab Statistics Toolbox.

Additionally, we have to account for the fact that the classes in our dataset are severely imbalanced with more non-events than events (at a ratio of 1:200 in our simulations). Such class imbalance can lead to problems for machine learning algorithms [27]. Therefore, we down-sample the majority class (*Y*^(*i*)^=0) to achieve a ratio of 1:3, yielding a good tradeoff between class balance and number of overall samples. Feature selection using Lasso is applied to this down-sampled dataset via Eq. (12). Since down-sampling intentionally discards data and Lasso feature selection is sensitive to data perturbation [25], we repeat the procedure *N*=50 times, each time using a different sample of the majority class, combining it with the minority class and fitting the Lasso to this dataset. This approach is adapted from rare event logistic regression with replication [28] and is reminiscent of bootstrap Lasso [29]. Finally, for each feature, we record the probability of inclusion in the model, i.e. the percentage of the *N* iterations that included the feature into the model at the optimal regularization strength *κ*^∗^. We consider those features to be relevant that have an inclusion probability larger than 90 % [29]. This yields the final set of features for our model. We now fit this sparse model to the full data without the *L*_1_ penalty (a process called “debiasing” [25]), since *L*_1_ regularization is biased towards too small weights. We thus obtain our final model, its associated weights $\hat w$ and the corresponding transition rate $\hat \lambda (t,F) = -\hat w^{T} F \cdot \Delta t$.

**Fig. 5:**
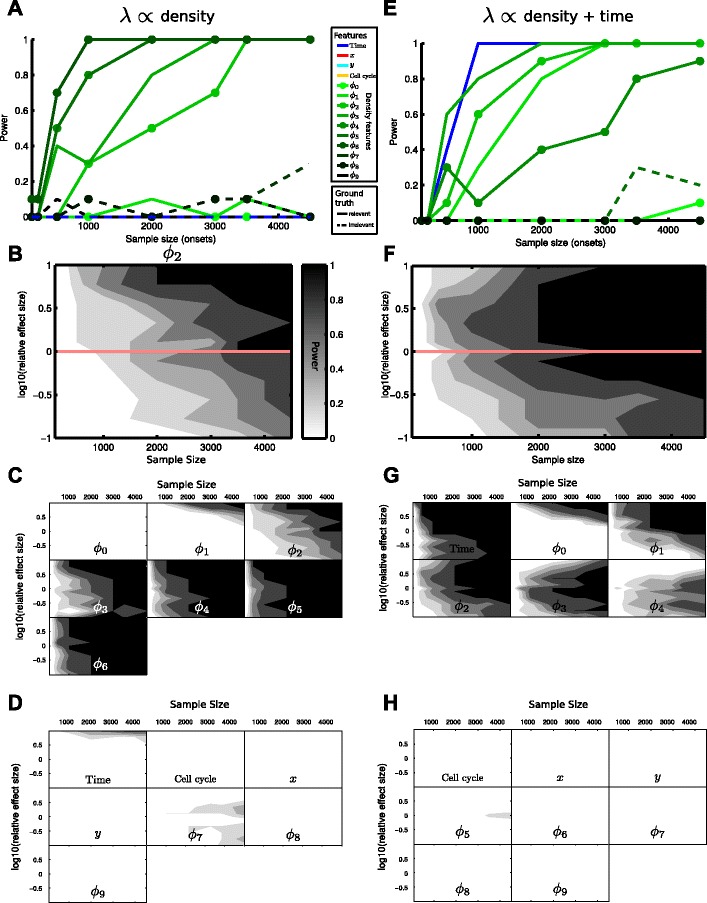
The method’s performance is robust for different sample size and effect size. **a** The statistical power for each feature (the probability of including the feature into the model) plotted against sample size (other parameters as in Fig. 4 a-c). Relevant features have high probability of being included in the model (power ≥0.8) when 2000 or more transition events (corresponding to approximately 44 genealogies) are used for the analysis. Solid (dashed) lines correspond to relevant (irrelevant) features in the respective scenario. **b** The power for the density feature *ϕ*_2_ shown as a function of sample size and relative effect size. The red line indicates the section corresponding to **a** with a relative effect size of 1. As expected, power increases with sample size and relative effect size. **c**,**d** The power as a function of sample size and relative effect size for all **c** relevant and irrelevant **d** features of the scenario. Colorbar as in **b**. **e**-**h** Analogous to **a**-**d**, but for a dataset used in Fig. 4 d-f, where the transition rate *λ* depends on time and local cell density with a Gaussian kernel

**Fig. 6:**
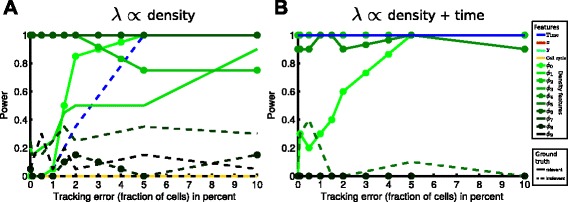
The method’s performance is robust for moderate amount of tracking error. **a** Statistical power plotted against the amount of tracking error for the density dependent scenario from Fig. 4 a-c (4500 onsets). Solid (dashed) lines correspond to relevant (irrelevant) features in the respective scenario. The correct features are identified reliably (power ≥0.8) up to a tracking error of 3 %. For larger tracking error, there is a high probability to include time (blue curve) into the model even though it is only an indirect influence. Note that tracking error seems to some extent facilitate the detection of *ϕ*_0_,*ϕ*_1_ (see main text for details). **b** Analogous to **a**, but for the dataset where the transition rate *λ* depends on time and local cell density with a Gaussian kernel (4500 onsets)

The inclusion probability threshold (0.9) controls the probability *α* of a type I error, i.e. including a feature even tough it is irrelevant. In addition it is also important to assess the probability *β* of type II errors, i.e. the chance that a relevant feature is not included into the model, or equivalently, the statistical power=1−*β*, which is the probability of discovering the feature if it is indeed relevant. The power is a function of sample size and effect size (the parameters *a* and *b* in Eqs. 6–8): The more samples are available and the larger the effect size, the higher to probability to discover a relevant feature. Since no analytical expressions are available, we estimate the statistical power of our model with respect to a certain feature through repeated simulation: Given a fixed sample size and effect size, we generate *M* independent datasets, apply the above GLM with bootstrapping-based feature selection to each dataset, resulting in *M* different models, which might have selected different features. We then approximate the statistical power as the fraction of the *M* models that correctly selected the feature of interest. Since computations become demanding (sample size and effect size/tracking error have to be varied, see Fig. 5), we choose *M*=10 (Fig. 5) and *M*=20 (Fig. 6).

### Expected frequencies of subtree patterns in cellular genealogies

**Fig. 7:**
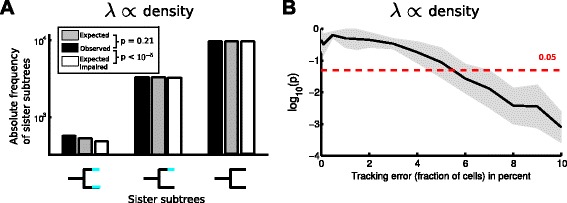
Expected frequencies of sister pairs reveal if the model can account for the observed genealogical correlations. **a** Comparison of the observed and expected frequencies of sister pairs (both, one, or none undergoing a state transition) of the dataset used throughout Fig. 4 a-c shows no significant difference (*p*=0.21, *χ* ^2^-test, see Methods). Fitting the same data, but not accounting for the *ϕ*_5_,*ϕ*_6_ features causes significant deviations from the expected frequencies (*p*=1.3·10^−6^). **b** P-values of the *χ* ^2^-test (average and standard deviation over 10 replicates) to compare the observed and expected frequencies of sister pairs against amount of tracking error for the density dependent scenario. For tracking errors <5 %, the method correctly concludes that the frequencies of observed sister pairs are in agreement with the model (applying a significance threshold of *α*=0.05, red dashed line)

Having estimated the transition rate $\hat \lambda $ via the regularized GLM, we calculate the number of subtree patterns expected under this transition rate. In the following we consider only subtrees of 1 generation, i.e. a mother and its two daughter cells, but the approach is easily extendable to larger subtree patterns. The expected frequencies of sister cell pairs where in either both cells, one cell, or none of the two cells state transition occurs, can be used to validate the inferred transition rate (see Fig. 7a and Additional file 1: Figure S1). We define the random variable *C*_*i*_ to indicate whether cell *i* underwent a state transition within its lifetime (*C*_*i*_=1) or stayed in state I (*C*_*i*_=0). Note that the *C*_*i*_ describe the state of a cell over its entire lifetime, as opposed to the *Y*^(*i*)^ used in the previous section, which denote the state of a cell at a small time interval *Δt*. Using the estimated transition rate $\hat \lambda $, we calculate the probability of a state transition in a single cell *i* as

(13) $$\begin{array}{*{20}l} P(C_{i}=1) = p_{i} = 1-\exp\left[- \int_{\zeta_{i}}^{\eta_{i}} ds\hat\lambda(s,F_{i}(s))\right] \end{array} $$

where $\hat \lambda (s,F_{i}(s))$ is the estimate of the transition rate the cell experiences throughout its lifetime [*ζ*_*i*_,*η*_*i*_] based on its features *F*_*i*_(*s*) (Additional file 1: Figure S1). Similarly, we derive the probability of a state transition in its sister cell *i*^′^ as $P(C_{i^{\prime }}=1)\phantom {\dot {i}\!}$. Considering the whole dataset containing *M* pairs of sister cells (*i*,*i*^′^),*i*=1…*M*, the expected number of pairs where both sister undergo a state transition is:

$$\begin{array}{*{20}l} E_{2} = \sum_{i=1}^{M} P(C_{i}=1, C_{i'}=1) \;, \end{array} $$

where $P(C_{i}=1, C_{i^{\prime }}=1)$ is the joint probability of these events. However, assuming independence between sisters, this factorizes to

$$\begin{array}{*{20}l} E_{2} &= \sum_{i=1}^{M} P(C_{i}=1) \cdot P(C_{i'}=1)= \sum_{i=1}^{M} p_{i} \cdot p_{i'}\;. \end{array} $$

The expected number of pairs where a state transition occurs in only one sister (*E*_1_) and in none of the sisters (*E*_0_) are:

$$\begin{array}{*{20}l} E_{0} &= \sum_{i=1}^{M} (1-p_{i}) \cdot (1-p_{i'})\\ E_{1} &= \sum_{i=1}^{M} (1-p_{i}) \cdot p_{i'} + p_{i} \cdot (1-p_{i'})\;. \end{array} $$

Applying Eq. 13, we can evaluate (*E*_0_,*E*_1_,*E*_2_) in terms of the estimated transition rate $\hat \lambda $.

In order to test whether our observed data matches these expected frequencies (*E*_0_,*E*_1_,*E*_2_) we count the observed frequencies (*O*_0_,*O*_1_,*O*_2_) in the data and perform a *χ*^2^ test with two degrees of freedom and

$$ \chi^{2} = \sum_{j=1}^{3} \frac{(E_{j}-O_{j})^{2}}{E_{j}}\;. $$

## Results

In the following, we use our generative model to simulate datasets from the simple cell state transition model (Fig. 2a) according to four different scenarios, where the transition rate *λ* depends on different features (e.g. a time dependence or cell density dependence). Subsequently, we apply our proposed inference methods to the data from different scenarios, assuming the data generating scenario is unknown. We show how the dependence of *λ* can be recovered from the data, e.g. allowing us to distinguish density- and time-dependent scenarios. Furthermore, we analyze the impact of sample size and tracking error on our results in order to assess the required experimental design.

### Estimating the transition rate non-parametrically from simulated data

In the simplest scenario the rate *λ* is constant during the whole time of observation (*λ* constant, Eq. 5). This corresponds to state transitions occurring spontaneously independent of other influences. Using our generative model for cellular genealogies (see Methods for details), we generate a sample of 100 genealogies with constant rate *λ*. We then reconstruct the rate $\hat \lambda $ from the data via Eq. 10 (black curve in Fig. 3a). The underlying true rate *λ* (red curve in Fig. 3a) is well contained within the Bayesian 95 % credibility intervals of our estimate (gray areas in Fig. 3a).

Next, we simulate 100 genealogies with a linear time-dependent transition rate (*λ*∝ time, Eq. 6). With the same approach we estimate $\hat \lambda $ (see Fig. 3b) and again, we observe good agreement between the estimated (black curve in Fig. 3b) and the true transition rate (red curve in Fig. 3b).

We now account for cell-cell communication and consider a transition rate depending on local cell density (*λ*∝ density, Eq. 7): the more cells present in the vicinity of the cell of interest, the more likely it is that a state transition occurs. We estimate the density dependent rate from 100 simulated genealogies, assuming we already know the underlying density kernel (this assumption will be relaxed later on). The estimated rate $\hat \lambda (\rho)$ (black curve in Fig. 3c) linearly increases with local cell density and the true rate is well contained within the credibility intervals (gray area in Fig. 3c), showing that one can identify the influence of local cell density on the transition rate. Note that the estimates of the transition rates at high density (*ρ*>35 in Fig. 3c) carry large statistical uncertainty (indicated by the broad credibility intervals) simply because very few cells are observed in those high local cell densities.

However, if we instead estimate the rate as a function of time from the same dataset, we would conclude that it is time-dependent, since the rate strongly increases over time (see Fig. 3c, inset). This is an indirect dependence: as time increases, local cell density grows exponentially and as a result, cells are more prone to undergo a state transition (see Fig. 3d inset). We can resolve this by estimating the rate simultaneously as a function of time and local density, $\hat \lambda (t,\rho)$ (Fig. 3d). For fixed local density *ρ*, the rate is almost constant across different times (black arrow in Fig. 3d). However, the transition rate changes considerably if the local density changes. Therefore, we can conclude that the true transition rate depends only on local cell density. Notice however that this conclusion relies on having sufficiently many samples, yielding a good coverage of the (*t*,*ρ*) space, and knowledge of the range (*R*) and nature of the spatial interaction. If *R* is chosen too small, any dependence of *λ* on the local cell density is hidden by the dominating indirect time-dependence. Moreover, analyzing $\hat \lambda $ visually becomes infeasible for higher feature dimensions.

### Estimating the transition rate with generalized linear models

To approach the aforementioned issues, we infer the transition rate more systematically using the machine-learning framework of generalized linear models (GLMs, see Methods for details). Instead of considering only one feature at a time, we include all features at once and apply feature selection to determine the relevant ones. An additional advantage of this approach is that it is not necessary to assume any density kernel a priori (as in the previous section). Instead, we use a set of spatial features *ϕ*_*k*_, whose linear combination can approximate any kernel (Eq. 4). We then use the proposed GLM equipped with *L*_1_ regularization to learn the relationship between features and class label and to obtain those features that directly influence the state transition rate.

We apply this approach to the density-dependent dataset (*λ*∝ density, Eq. 7). Starting with strong regularization (that is, a large *κ* and consequently a sparse model) only the most relevant features have non-zero weights and are included (Fig. 4a). By decreasing the regularization parameter, the weights of the features gradually increase, making the model more complex. The optimal regularization *κ*^∗^ (the black line in Fig. 4a corresponds to the mean of *κ*^∗^ across the 50 bootstraps) is determined by cross validation (see Methods for details). All features with non-zero weights at *κ*^∗^ are included in the model. The ground truth of features used to simulate the dataset is indicated by solid (relevant) and dashed (irrelevant) lines in Fig. 4a.

We estimate the inclusion probability of a feature as the fraction of the 50 bootstraps that selected the feature (Fig. 4b). For example, the features *ϕ*_2_,…,*ϕ*_6_ (representing local cell densities at different radii, see Fig. 2d) are present in all bootstraps, *ϕ*_8_ is present in 70 % of the bootstraps, and all other features have low inclusion probabilities. In particular, time is included in only 18 % of the bootstraps and spatial location (x,y) and time since last division (cell cycle) have zero inclusion probability. Choosing a cutoff at 90 % (gray area in Fig. 4b) for a feature to be included in the final model, we recover all features (except *ϕ*_0_,*ϕ*_1_) that were used to generate that dataset. We miss *ϕ*_0_ and *ϕ*_1_ since their contribution to the overall transition rate is effectively very low: the average number of cells within *ϕ*_1_ is approximately 0.2, whereas the average number of cells within e.g. *ϕ*_7_ is approximately 1. Hence, leaving out *ϕ*_1_ will not change the overall result, and the algorithm chooses to neglect the feature in favor of sparsity.

After feature selection, we can reconstruct the density kernel as a weighted sum of the basis functions *ϕ*_*k*_ via Eq. 4 (shown as green bars in Fig. 4c). Here, we observe that the reconstructed kernel closely resembles the true underlying tophat kernel that was used to simulate the data (shown as a black curve in Fig. 4c). To demonstrate that the method can faithfully report the range of the spatial interaction, we performed the same analysis on a dataset with a density dependence mediated via a short range tophat kernel (*R*=40 *μm*), which indeed can be recovered from the data (Additional file 1: Figure S2).

We extend the set of relevant features and now consider a scenario where the transition rate depends on time and on local cell density (*λ*∝ density + time, Eq. 8), this time modeled via a Gaussian kernel (with *σ*=130 *μm*) instead of a tophat kernel to illustrate the versatility of our method. Since the Gaussian kernel has infinite support, a priori there is no clear definition which *ϕ*_*i*_ are relevant. In the following, we define all *ϕ*_*i*_ inside the 95 % quantile of the Gaussian distribution as relevant. This results in *ϕ*_0_,…,*ϕ*_4_ considered relevant while *ϕ*_5_,…,*ϕ*_9_ are deemed irrelevant.

The regularization path and the feature inclusion probabilities (Fig. 4d, e) show that the GLM correctly selects both time and local cell density (*ϕ*_1_,…,*ϕ*_4_) with inclusion probabilities close to 1. Finally, using the weights associated with the selected density features we reconstruct the kernel of local cell density and find that it indeed matches a Gaussian kernel (Fig. 4f). As before (Fig. 4a-c), the feature selection procedure misses *ϕ*_0_ due to its relatively small contribution to the overall transition rate. We conclude that our proposed method is capable of identifying the features that are most predictive of the transition rate and faithfully filters out indirect influences. Furthermore, we can estimate the shape of the density kernel from the data.

### Sample size, effect size and statistical power

Accurate single-cell identification and tracking in time-lapse movies is still a challenging task and requires, at least in mammalian systems manual data curation [12, 13]. Thus estimating the required sample size for any given effect size is necessary for efficient experimental design.

To assess the impact of sample size on the performance of the feature selection, we systematically reduce the number of observed state transition events (by reducing the number of genealogies) and calculate the statistical power of our method, i.e. the probability to detect a certain effect if present in the data (Fig. 5a, e). Starting at the original sample size of 4500 onsets (using all 100 genealogies), we find that the power is 1 for the features detected previously (Fig. 4b, e), suggesting these features can reliably be detected. Similarly, the model’s power with respect to features *ϕ*_0_ and *ϕ*_1_ is 0, hence those features are not detectable at the given sample size. Decreasing the sample size, the power for certain features gradually drops (e.g. *ϕ*_2_ in Fig. 5a): The data no more contains sufficient statistical information to identify the feature as relevant. At a sample size below 1000 events, the power for all features is considerably smaller than one such that non of the features can reliably be identified. However, a sample size of 2000 onsets (corresponding to 44 genealogies) is sufficient (based on the established threshold of power >0.8) to faithfully detect the most important features influencing the transition rate and to distinguish a direct time-dependence (Fig. 5a) from an indirect one (Fig. 5e).

The statistical power does not only depend on the available sample size but also on the strength of the effect, i.e. small effects will be hard to detect for a fixed sample size than a strong one. We therefore vary the effect strength by changing the parameters *a* and *b* in Eqs. 7, 8 within one order of magnitude and estimate the power for each feature not only as a function of sample size but also of effect strength (relative to our baseline scenarios used in Fig. 4). As expected the power increases with increasing sample size and effect strength. For example, in the density dependent scenario, for a large relative effect size of 10, 1500 samples are sufficient to yield a power of 0.8 for feature *ϕ*_2_, while for small effect size (0.1) more than 4000 samples are needed to achieve the same power (Fig. 5b). Furthermore, features *ϕ*_3_,…,*ϕ*_6_ can reliably be identified (power >0.8) with more than 2000 onsets almost independent of the effect strength considered (Fig. 5c). In contrast, *ϕ*_0_ cannot be detected (power =0) for any of the given effect strengths and sample sizes, and *ϕ*_1_,*ϕ*_2_ are only detectable for both large effect size and sample size (Fig. 5c).

Looking at the irrelevant features (Fig. 5d), the probability of detecting them as relevant is mostly zero and they are correctly eliminated from the model. Only for large effect size, ‘Time’ has non-zero probability of being contained in the model: Due to the large effect size, state transitions happen after the first cell division (the transition rate increases strongly as soon as one daughter senses the presence of the other) and hence time- and density-dependence cannot be distinguished.

For the dataset used in Fig. 4d-f, where the transition rate *λ* depends on time and local cell density with a Gaussian kernel, similar patterns are observed (Fig. 5f-h). Time is identified reliably (power >0.8) for a sample size large than 1500 onsets, while for the other relevant features (*ϕ*_0_,…,*ϕ*_4_) more samples or a larger effect size are needed (Fig. 5g). Interestingly, larger effect size seems to decrease the power for features *ϕ*_3_,*ϕ*_4_ (Fig. 5g). If the effect size is very large, most state transitions will happen even before cells spread out in space such that the outer density features get populated. Therefore their effect cannot be inferred from the data.

### Influence of tracking error

To obtain genealogies from time-lapse microscopy data, manual [Schwarzfischer et al., in revision] or automatic tracking (for an overview of current methods, see [30]) is required. Neither automatic nor manual tracking can produce perfect genealogies, but will introduce errors especially when local cell density is high or cells move fast as compared to the time resolution of the imaging. To test the influence of tracking errors on the our method, we introduce artificial tracking errors into the simulated datasets by interchanging the identity of randomly selected cells of the same generation and hence swapping entire subtrees of the genealogies. The amount of tracking error is defined as the percentage of all cells in the dataset where an artificial tracking error was introduced. We simulate different amounts of tracking error with up to 10 % of all cells in the experiment containing a tracking error. Note that tracking errors impact our analysis only by the creation of spurious state transitions (a cell in state I is at some point accidentally interchanged with a cell in state II). We now evaluate the previous results on these erroneous datasets.

We find that for both the density-dependent (*λ*∝ density, Fig. 6a) and the time- and density-dependent scenarios (*λ*∝ density + time, Fig. 6b) our method reliable identifies the underlying features (power ≥0.8) for up to 3 % of tracking error. For higher amounts of tracking error, we erroneously identify time as a relevant feature and fail to identify *ϕ*_2_ as relevant feature in the first scenario (blue line in Fig. 6a). Note that the wrong inclusion of time is due to the fact that tracking errors and the spurious state transitions created by those errors are more likely at later timepoints where more cells are present. Hence, those spurious onsets at late timepoint lead to the inclusion of time into the model.

For the second scenario (Fig. 6b), identification of the relevant features seems to be very robust with respect to tracking error, as all of them have power >0.8 even for 10 % tracking error.

In both scenarios, tracking error seems to facilitate the detection of *ϕ*_0_ (and *ϕ*_1_ for the density dependent scenario) that was not detectable previously or only for large effect size (see Fig. 5c, g). As discussed before, *ϕ*_0_ is removed by the Lasso in favor of sparsity as the other features are sufficient to explain the data. Tracking error increases the noise level, i.e. the correlation between relevant features and class labels *Y*^(*i*)^ becomes weaker. Since the other features are no longer sufficient to explain the transition events, the Lasso includes *ϕ*_0_, which now significantly improves the model.However, at some point tracking error and hence the noise level will become so large that relevant features become decorrelated with the events and LASSO removes them again in favor of sparsity.

### Model validation using sister correlations

Apparently, our method is able to infer state transition mechanisms by identifying relevant features even in the presence of moderate tracking errors. However, what if we miss to include relevant features in the GLM, e.g. unobserved influences like nutrient concentrations? In this section, we show how to use the tree structure – if available – to validate the chosen model. We investigate whether the transition rate *λ* estimated by the GLM is capable of explaining the observed correlated transition events within the cellular genealogies. We focus here on correlations between sister cells, but the approach easily generalizes to higher order relationships within a genealogy, like cousin-quartets. Suppose that we obtained a reasonable estimate $\hat \lambda $ of the transition rate. Then, the state transition of one sister cell is independent of the other and just determined by the transition rate that might differ due to the spatial context in both cells. With this independence assumption, we can calculate the probability to observe sister subtree patterns (where both, one or none of the sister cells change state) just as the product of the individual probabilities (see Methods). Note that these probabilities are calculated over the entire lifetime of each cell finally resulting in the expected number of sister subtree patterns for the entire dataset.

Using these frequencies, we assess if the transition rate estimated by the GLM (agnostic of the tree structure) is capable of explaining the observed correlations in the genealogies and therefore is an adequate description of the data. For the dataset where the state transition depends only on local cell density (*λ*∝ density, Eq. 7), we calculate the expected frequencies of sister subtrees given the previously estimated transition rate (Fig. 7a, gray bars) and compare these to the observed frequencies in the data (Fig. 7a, black bars). No significant differences are observed (*p*=0.21, *χ*^2^-test, see Methods), and hence, there is no indication of correlations beyond what we expect from the density dependent transition rate, in agreement with the generative model.

Next, we show how this idea can be used to determine if all relevant features have been included in the GLM. To that end, we now deliberately neglect the spatial features *ϕ*_5_,*ϕ*_6_ when fitting the transition rate via the GLM. Since these two features influence the transition rate in the chosen scenario, fitting the impaired GLM yields a different $\hat \lambda $ and hence also different expected frequencies of sister subtrees (Fig. 7a white bars). The frequencies are significantly different (*p*=1.3·10^−6^), indicating the model is inappropriate, as there is more correlation in the trees than the model can account for (due to the missing *ϕ*_5_,*ϕ*_6_). This difference is most pronounced for the pattern where both sister cells change their state.

Furthermore we performed this analysis for a smaller sample size with 2000 onsets (which are sufficient to recover the most relevant features, see Fig. 5a) and recover a similar result (see Additional file 1: Figure S3): While observed and expected frequencies of sister subtrees are not significantly different, impairing the model leads to significant differences in the sister subtree frequencies.

Our approach to validate the model using sister correlations (Fig. 7a) relies on entire correct trackings of both sister cells, as we integrate over the entire lifetime of these cells in Eq. 13. Analogous to Fig. 7a, we evaluate whether we observed frequencies of sister subtrees match the expectations of the model (which was also fitted to the dataset containing the tracking errors) via a *χ*^2^-test for different amounts of tracking error. For the density dependent scenario, we find that up to 5 % of tracking error, we do not observe significant differences between observed and expected frequencies (*α*=0.05), correctly indicating that the density dependent transition rate can explain the observed frequencies (Fig. 7b). However, more than 5 % of tracking error result in substantial changes of the sister correlations, which cannot be explained by the model of the transition rate (shown by the significant differences in frequencies).

## Discussion

In this paper, we have presented a method to investigate mechanisms driving cell state transition events observed in cellular genealogies. As two features explicitly regulating the transition rate, we have here considered time and local cell density. Our method is complementary to the approach by Snijder et al. [31] who showed that the response of a cell to a certain stimulus (in their case, a virus infection) strongly depends on each cell’s “population context”, that is, its localization within the colony, its cell density and cell cycle stage. This approach, which has been applied to the analysis of high-content screens by Knapp et al. [32], is designed for static data and a single, controlled perturbation. The cells are subject to a treatment at a defined timepoint and their response is recorded by a single image. For our purpose a static approach, where the timepoint of the event is predetermined, is not applicable. Instead, we assume that cells undergo state transitions spontaneously, and hence transition events can happen at any point in time but their probability chances over time due to the changing environment the cells experience.

Our method currently assumes a linear relationship between features and the transition rate (see Eq. 11). Hence, it is necessary to discuss whether the model can recover relevant features in the presence of nonlinearities or how it can be adapted. In general it is difficult to predict the outcome when fitting a GLM that assumes a linear transition rate to data generated with a nonlinear transition rate. On the one hand, performance might suffer as the model cannot capture the nonlinearities and might potentially select the wrong features. On the other hand, nonlinearities might simplify the task of identifying relevant features. For example, if the transition rate is a steep, sigmoid function of cell density, this influence will be easier to detect than a linear one: In feature space, the samples with transition events (*Y*^(*i*)^=1) will be clearly separated from the samples without events (*Y*^(*i*)^=0) in the nonlinear case, while in the linear case there’s a continuum and no clear separation between those two classes. We simulated a scenario where the transition rate is a sigmoid function of cell density (Additional file 1: Figure S4). Here, our method can still deduce the relevant features despite the nonlinear relation. More generally, one can extend the presented method to handle nonlinearities: The set of features *F* can be augmented by nonlinear transformations, e.g. by including quadratic or interaction terms (e.g. ${\phi _{i}^{2}}, \phi _{i}\phi _{j}$) into the data matrix and feature selection is performed on this extended set. Alternatively, the GLM can be replaced by nonlinear classifiers, e.g. random forests [33]. While those methods can handle nonlinear relationships in the data, they lack the build-in feature selection of LASSO and will in general not be sparse. For random forests, one can instead use the calculated feature importance measures to perform feature selection.

In our model, we assume that cells can undergo just a single fate transition (black cells turn into blue cells, Fig. 2a), while for example in stem cells, fate decisions are often binary, i.e. cells have to choose between two mutually exclusive follow-up states. For illustration, let us assume that black cells turn either into blue or red cells. The proposed method can easily be adapted to this setting. Two different scenarios should be distinguished: 1) The two transitions are entirely independent, i.e. there exist two separate transition rates *λ*_1_,*λ*_2_ and whatever fate is chosen first determines the resulting cell state [34]. In this case, one can simply split the dataset into the cells undergoing the one transition and those cells undergoing the other transition and fit the model to both sets separately. 2) The transition time is determined by a single transition rate *λ*, and the outcome (blue vs red cell) is determined by a probability *p*, which might again be a function of features such as cell density. Here, one would first build a model for the transition rate *λ* (simply treating red and blue cells alike) and in a second step build a model of *p* (considering now only blue and red cells). If it is unknown which setting applies a priori, both have to be analyzed and later compared to determine which one best explains the data.

Our model assumes a homogeneous cell population, i.e. all cells in state I (Fig. 2a) are equivalent and share the same transition rate. In reality however, apparently homogeneous cell populations often contain subpopulations that behave differently but cannot be distinguished a priori by e.g. surface markers. In our context one could imagine two subpopulations of cells in state I: One subpopulation that undergoes state transitions in response to cell density (i.e. the transition rate is a function of cell density), while the other subpopulation obeys a transition rate that is a function of time. In our model that is unaware of the subpopulations, two potential results can be imagined: The model might identify time and cell density as relevant features but it will miss the fact that cells respond to either one of those features. Alternatively, the model might consider both density and time as irrelevant as neither of them is capable of explaining all the observed data, but just a fraction of it. Here, one has to use more flexible models than a GLM. A natural choice are “Mixtures of generalized linear models” [35], where instead of fitting a single GLM to the data, multiple GLMs are fitted which are responsible for different parts of the data. Ideally this would result in a mixture model with two GLMs, one containing only density features, the other containing only time as relevant variables.

In time-lapse microscopy, the cell’s state is usually read out via surface markers. We here assume that a change in such surface marker expression reports a cell state transitions immediately. However, the marker might not be perfect, i.e. if the cell undergoes the transition but the marker changes only several hours later causing a delay between the event and its observation. If this delay is short relative to the autocorrelation time of the relevant features (e.g. if the cell density a few hours after the state change is still comparable to the density at the transition), our proposed method is still capable of detecting the effect. However, delays becomes more difficult to handle in the same way that tracking error degrades the performance: The noise level increases and decorrelates predictors and response variables. A much longer delay (e.g. several generations) might be caused by cell intrinsic processes, e.g. a new gene expression program is initiated after the state transition and a change in phenotype (the upregulation of a marker gene) is observed only once this program has been completed. This causes correlations between related cells that cannot be explained by the observed features (see ‘Model validation using sister correlations’). Here, one has to model the delay explicitly, exploiting the fact that the particular correlations between the cells inform about the delay length: For example, if one observes strong correlations between sister cells, but no correlations between cousins, this would indicate a delay of about one generation.

Note that our approach shares certain aspects with Cox proportional hazard models [36]. The standard Cox proportional hazards models use fixed covariates measured once per individual to predict the time to an event for that individual. However, they can be extended to account for time varying covariates (measured several times per individual) using the counting process reformulation by Andersen and Gill [37]. Analogous to our approach, one then considers small time intervals where the covariates are constant and builds a model that predicts whether an event happened in these small intervals. This reformulation is also crucial for our approach as it allows to handle the tree structure of the data by dissecting it into small intervals. The main difference of our model to proportional hazards is the form of the hazard rate (in our context the transition rate), which in our case is linear in the covariates (see Eqs. 6–8), while in the Cox proportional hazard model it is multiplicative in the covariates. This choice is motivated by the form of the transition rate in an earlier study [7]. In general our assumption is equally strong as the proportional hazards assumption, however not relying on the proportional hazards machinery is beneficial as one then can easily exchange the GLM framework by alternative machine learning techniques if required.

In the current formulation, we assume that the transition rate of a cell *i* at time *t* is a function only of the features *F*_*i*_(*t*) at time *t* (the transition event is a point process) and not a function of the history *F*_*i*_(*s*),*s*<*t* of the cell. On the one hand this is advantageous because no extensive tracking of cells over multiple generations, but only an accurate cell segmentation at time *t* is required to assess all observable features *F*_*i*_(*t*) such as cell density. On the other hand, the method cannot identify a history-dependence of the transition rate, e.g. in a scenario where a cell integrates over the previously experienced cell densities via some internal mechanism. However, given reliable single cell tracking data and hence reliable timecourses of the features *F*_*i*_(*t*) for single cells *i* instead of snapshots, the presented approach can be extended to also detect history dependent transition rates. To that end, one has to augment the feature vectors entering the GLM by time-shifted versions of those features, i.e. including not only present cell density, but also densities at previous timepoints, and fits the model as proposed. This is analogous to applying the idea of Granger causality [38] to feature timecourses *F*_*i*_(*t*) and binary cell state timecourses *Y*_*i*_(*t*), i.e. inferring what features (including their history) contain information to predict the state-timecourses. Accounting for potential history-dependence with time-shifted versions of the original features complicates the inference problem due to the increasing feature space, potentially requiring a larger sample size. However, the following reasons might still allow for a faithful inference: (i) The Lasso regularization with bootstrapping is known to work well even for high dimensional problems [29]. (ii) Since the features have considerable autocorrelation (local cell density does not change rapidly over time for typical cell speeds), one only has to include the time-shifted features at intervals greater than the autocorrelation time. This leads to fewer features than considering every possible time-shift for each original feature. Still, if the transition rate depends on a very long history (e.g. several generations) this approach might become infeasible due to the increasingly large features space. Here one has to augment the problem with some additional regularization, e.g. enforcing the weights of neighboring time-lags to be similar [39].

We showed how the kernel for spatial interactions can be learned from the data using a set of concentric basis functions with width *Δr* controlling the resolution of the kernel. From a biological perspective the most interesting quantity of the interaction kernel is its range, i.e. the distance on which cells communicate and influence each other. A kernel range on the order of a typical cell size indicates that state transitions are initiated or inhibited due to cell-cell contact (e.g. Delta-Notch signaling [40]). Large kernel range suggest communication via signaling molecules, e.g. cytokines that are able to instruct cell fate choice in stem- and progenitor cells [17, 41]. This range can be inferred using a relatively coarse kernel resolution (large *Δr*, also see Additional file 1: Figure S2). A fine resolution (small *Δr*) has to be chosen if the precise shape of the kernel is of interest. From the exact shape of the kernel one could learn about the signaling mechanism, e.g. how the signal is integrated by the receiving cells. For example, a long range tophat kernel would indicate a threshold response in the signal-receiving cell, i.e. the cell’s surface receptor transfers the signal into the cell only if the signaling molecule exceeds a certain concentration. A long range Gaussian kernel would instead indicate that the receiver responses gradually to the signal. However, a fine kernel resolution comes at the expense of a more challenging inference problem: Not only does the number of features *ϕ*_*i*_ increase (if the same overall range of interactions has to be covered) but more importantly, the contributions of the individual *ϕ*_*i*_ becomes weaker such that they are more likely eliminated by the Lasso regularization (analogous to *ϕ*_0_,*ϕ*_1_ in Fig. 4a-c). This can either be compensated by increasing the sample size or putting constraints on the weights of the *ϕ*_*i*_, e.g. enforcing the weights of neighboring *ϕ*_*i*_ to be similar, resulting in smooth kernels (similar to fused lasso [39]).

With respect to regulating features, our method can be extended to any other parameter that is experimentally accessible. In terms of tumor growth for example, the presence (or local density) of distinct cancer cell subtypes might influence transitions between states of different proliferative potential [42]. This could be analyzed by introducing cell type specific density features ${\phi _{i}^{c}}$ that take into account only a certain cell type *c* when calculating local cell density. For blood progenitor cells, including the expression levels of Pu.1 [43], a pivotal fate determining factor [44], as a feature will allow to compare extrinsic and intrinsic [45] effects on cellular plasticity.

## Conclusion

Our approach is designed for dynamic data provided by time-lapse microscopy, which allows to observe state transitions in their spatiotemporal and genealogical context. The requirements for an appropriate dataset are (i) single-cell genealogies obtained from automatic or manual cell tracking, (ii) at least as many annotated state transitions as determined by our analysis, and (iii) the identification of all cells surrounding a transition event in an sufficiently large radius. To the best of our knowledge, no such dataset exist up to now, but manual and automated tracking tools increase accuracy and efficiency ([13, 46]; [Schwarzfischer et al., in revision]). Moreover, our method relies only on short trackings of one cell cycle to quantify sister correlations (Fig. 7). Since fluorescent fate markers exist for various systems, morphological quantification has been shown to be usable for fate recognition [47], and robust cell segmentation algorithms work on full time-lapse movies [16], we believe that adequate datasets from various cell systems will emerge in the near future. Due to the method’s generality, many different types of cell state transitions can be investigated in their spatiotemporal context: For example, one can study the influence of cytokine signaling between differentiating blood stem- and progenitor cells [17], i.e. if the presence of one celltype (potentially secreting the cytokine) promotes specific differentiation decisions. In mouse embryonic stem cells the impact of cell-cell interactions on transitions between Nanog-high and Nanog-low cells [48, 49], or on the cell fate decision between epiblast and primitive endoderm [50] could be analyzed with our proposed method. Similarly, transitions between cancer stem cells and non-tumorigenic cells [51], or the epithelial-mesenchymal transition, which is thought to initiate tumor metastases [52] can be analyzed in their the spatiotemporal context.

## Additional file

Additional file 1
**Supplementary Text.** This document provides a detailed description of the full probabilistic model, Bayesian credibility intervals, the proof of sample independence, and the relation of log-binomial and Poisson regression. (PDF 345 kb)
