# Supplementary material for: Inference of spatiotemporal effects on cellular state transitions from time-lapse microscopy
Source: BMC Syst Biol. 2015 Sep 21;9:61. doi: 10.1186/s12918-015-0208-5 (PMC4578671; doi:10.1186/s12918-015-0208-5)
Supplement: Additional file 1 — Supplementary Text. This document provides a detailed description of the full probabilistic model, Bayesian credibility intervals, the proof of sample independence, and the relation of log-binomial and Poisson regression. (PDF 345 kb) [file 12918_2015_208_MOESM1_ESM.pdf]

Supplements: Inference of spatiotemporal effects on cellular state transitions from time-lapse microscopy

Michael K. Strasser, Justin Feigelman, Fabian J. Theis and Carsten Marr

July 24, 2015

1 The master equations of an interacting population of dividing cells

We consider a population of cells, each defined via its position $x \in \mathbb{R}^2$, its state $Y \in [0, 1]$ and its age $\tau \in \mathbb{R}^+$. Cells change state Y with rate $\lambda(F)$, where F are the cell's features (cell density, age, etc.), and divide with age dependent rate $\gamma(\tau)$. First, let us describe how the distribution of a single cell evolves:

$$\begin{aligned}\dot{\mathcal{P}}(x, Y, \tau, t) &= \nabla^2 \mathcal{P}(x, Y, \tau, t) + \frac{\partial}{\partial \tau} \mathcal{P}(x, Y, \tau, t) \\ &- \delta_{Y,0} \cdot \lambda(x, t, \tau) \cdot \mathcal{P}(x, Y, \tau, t) \\ &+ \delta_{Y,1} \cdot \lambda(x, t, \tau) \cdot \mathcal{P}(x, Y - 1, \tau, t) \\ &- \gamma(\tau) \cdot \mathcal{P}(x, Y, \tau, t)\end{aligned}$$

where $\delta_{n,m}$ is the Kronecker delta and $\nabla^2 = \frac{\partial^2}{\partial x_1^2} + \frac{\partial^2}{\partial x_2^2}$. The first term on the right hand side accounts for spatial diffusion of the cell in and out of location x, the second term accounts for aging, the third term accounts for cells transitioning out of state A ($Y = 0$) and the fourth term describes cells transitioning into state B ($Y = 1$). The last term accounts for the loss of the single cell due to cell division giving rise to a pair of cells.

When considering pairs of cells, we must describe the evolution of their joint distribution $\mathcal{P}(x_1, Y_1, \tau_1, x_2, Y_2, \tau_2, t)$:

$$\begin{aligned}\dot{\mathcal{P}}(x_1,&Y_1, \tau_1, x_2, Y_2, \tau_2, t) = \left(\nabla^2_{x_1,x_2} + \frac{\partial}{\partial \tau_2} + \frac{\partial}{\partial \tau_2} \right) \cdot \mathcal{P}(x_1, Y_1, \tau_1, x_2, Y_2, \tau_2, t) \\ &- [\delta(Y_1) \cdot \lambda(F_1(x_1, \tau_1, x_2, \tau_2)) + \delta(s_0) \cdot \lambda(F_2(x_1, \tau_1, x_2, \tau_2))] \cdot \mathcal{P}(x_1, Y_1, \tau_1, x_2, Y_2, \tau_2, t) \\ &+ \delta(Y_1 - 1) \cdot \lambda(F_1(x_1, \tau_1, x_2, \tau_2)) \cdot \mathcal{P}(x_1, Y_1 - 1, \tau_1, x_2, Y_2, \tau_2, t) \\ &+ \delta(Y_2 - 1) \cdot \lambda(F_2(x_1, \tau_1, x_2, \tau_2)) \cdot \mathcal{P}(x_1, Y_1, \tau_1, x_2, Y_2 - 1, \tau_2, t) \\ &+ \delta(\tau_1) \cdot \delta(\tau_2) \cdot \delta(x_1 - x_2) \cdot \int_0^t d\tau' \gamma(\tau') \mathcal{P}(x_1, Y_1, \tau', t) \\ &- [\gamma(\tau_1) + \gamma(\tau_2)] \cdot \mathcal{P}(x_1, Y_1, \tau_1, x_2, Y_2, \tau_2, t)\end{aligned}$$

The first line represent diffusion in space and drift in time. The second line corresponds to loss due to a state change out of state A, where the rate λ depends on the features F_i of cell i, which is function depending on potentially all system variables (e.g. cell locations when representing cell density). The third line accounts for a cell in state A ($Y_1 = 0$) at position x_1 that transitions into state B (the forth line is analogous for the second cell). The fifth line includes the gain of probability due to a division event in the single cell equation creating two cells of age 0. This term gives rise to the coupling of the equations. For simplicity we assumed that a division event

at location x gives rise to two cells both located at x as well. Finally, the last term models loss due to either cell 1 or cell 2 dividing.

The equation for higher numbers of cells (triplets, etc.) become more and more complex as one has to deal with arising symmetries (see e.g. Dodd and Ferguson [1]).

2 Credibility intervals for proportions from counting

When estimating the probability $P(t, t+dt|F_i(t))$ in Equation (9) of the main text, we in fact we estimate the parameter of a binomial distribution: we observed that k successes (events) occurred out of n trials and we are interested in the probability p of the success. Clearly, k follows a binomial distribution with parameters n, p:

$$k \sim \text{Binomial}(n, p)$$

Having observed a particular k and n we want to infer the parameter p of the underlying binomial distribution. In a maximum likelihood setting, one can show that this is just $\hat{p} = k/n$. Confidence intervals for this maximum likelihood estimator can be constructed according to various methods (Wald-, Wilson-, or Clopper-Pearson confidence intervals, [3]).

We invoke a Bayesian approach instead: using an uninformative conjugate prior for p (which is a Beta(1/2,1/2) distribution called Jeffreys prior, see [2]), we get the following posterior distribution for p:

$$P(p|k, n) \sim \text{Beta}(1/2 + k, 1/2 + n - k) \tag{1}$$

If we consider the posterior mean $\bar{p}$, we find

$$\bar{p} = \frac{k + 1/2}{n + 1}$$

which is approximately the same as the maximum likelihood estimate (k/n) if k, n are large. We obtain credibility intervals by calculating the 5% and 95% quantiles of the posterior in Equation (1).

A particularly appealing property of Bayesian credibility intervals is that they will strictly be within $[0, 1]$, unlike their frequentist counterparts which are not constrained to the $[0, 1]$ domain of probabilities. This is especially prevalent if the estimated probability itself is close to 0 or 1.

3 Independence of samples

For the machine-learning, we rely on the fact that the individual samples are independent. In the following we show why this is indeed true. Intuitively, one would think that for example two samples generated from the same cell, but at different timepoints within that cell are not independent: not only is there a strong correlation with respect to their features (e.g. because spatial position will change only slightly with time, and therefore the latter sample will be at a similar position to the earlier sample) but it seems that also the class labels are apparently not independent. For example the knowledge that the later sample is undifferentiated immediately implies that also the previous example is undifferentiated. However, this is not the case. Consider for example a single cell, that is created at time t_0 and differentiates at time t_N. The likelihood of this whole cell in terms of the hazard is

$$L = \lambda(t_N) \cdot e^{-\int_{t_0}^{t_N} d\tau \lambda(\tau)} \tag{2}$$

If we however decide to split up this cell into N individual observations O_i (as we do in the main text) we get the following likelihood for each observation:

$$\tilde{L}(O_i) = \begin{cases} e^{-\int_{t_{i-1}}^{t_i} d\tau \lambda(\tau)} & i \neq N \\ \lambda(t_N) e^{-\int_{t_{N-1}}^{t_N} d\tau \lambda(\tau)} & i = N \end{cases}$$

Assuming independence, the overall likelihood is

$$\begin{aligned}
\tilde{L} = \prod_i L(O_i) &= e^{-\int_{t_0}^{t_1} d\tau\lambda(\tau)} \cdot e^{-\int_{t_1}^{t_2} d\tau\lambda(\tau)} \cdot \ldots \\
&\ldots \cdot e^{-\int_{t_{N-2}}^{t_{N-1}} d\tau\lambda(\tau)} \cdot \lambda(t_N) e^{-\int_{t_{N-1}}^{t_N} d\tau\lambda(\tau)} \\
&= \exp\left[-\int_{t_0}^{t_1} d\tau\lambda(\tau) - \ldots - \int_{t_{N-1}}^{t_N} d\tau\lambda(\tau)\right] \cdot \lambda(t_N) \\
&= \exp\left[-\int_{t_0}^{t_N} d\tau\lambda(\tau)\right] \cdot \lambda(t_N)
\end{aligned}$$

Comparing this expression to Equation (2), we find that they are actually the same. Therefore the assumption of independence is true.

4 Log-binomial regression and its approximation via Poisson regression

In our simulations a single sample $(Y^{(i)}, F^{(i)})$ is created according to

$$\begin{aligned}
Y^{(i)} &\sim \text{Bernoulli}(p^{(i)}) \\
p^{(i)} &= 1 - \exp\left[-\lambda(F^{(i)}) \cdot \Delta t\right] \\
&= 1 - \exp\left[-w^T F^{(i)} \cdot \Delta t\right]
\end{aligned}$$

If we switch class labels such that

$$\begin{aligned}
V^{(i)} &= \begin{cases} 1, & Y^{(i)} = 0 \\ 0, & Y^{(i)} = 1 \end{cases} \\
V^{(i)} &\sim \text{Bernoulli}(q^{(i)}) \\
q^{(i)} &= 1 - p^{(i)} = \exp\left[-w^T F^{(i)} \cdot \Delta t\right] \; ,
\end{aligned}$$

we see that the generative model corresponds to a GLM with a Bernoulli distribution and an exponential mean function

$$E[V^{(i)}] = q^{(i)} = e^{-w^T F^{(i)} \Delta t} \tag{3}$$

This GLM is known as the log-binomial model and suffers from convergence issues as it allows probabilities greater than 1 (if the exponent in Equation 3 becomes positive). Hence, we choose to approximate the Bernoulli distribution by a Poisson distribution and keep the desired exponential mean function. This GLM is also known as Poisson regression [4]. Since the probability mass function of Bernoulli and Poisson distribution are very similar if $p \ll 1$, this provides a good approximation to the log-binomial model in the case of rare events:

$$\begin{aligned}
P_{\text{Ber}}(Y = 0|p) &= 1 - p \\
P_{\text{Ber}}(Y = 1|p) &= p \\
P_{\text{Poi}}(Y = 0|p) &= \exp(-p) \approx 1 - p \\
P_{\text{Poi}}(Y = 1|p) &= p \cdot \exp(-p) \approx p(1 - p) \approx p
\end{aligned}$$

The loglikelihood of the model (Equation (11) in the main text) for data (Y, X) and weights w is obtained as:

$$\begin{aligned}\log p(Y|F,w) &= \sum_i \log[p(Y^{(i)}|F^{(i)},w)] \\ &= \sum_i \log\left[\frac{\alpha^{Y^{(i)}}\cdot\exp(-\alpha)}{Y^{(i)}!}\right] \\ &= \sum_i \left[Y^{(i)}\log(\alpha) - \alpha - \log\left(Y^{(i)}!\right)\right] \ ,\end{aligned}$$

where we plugged in the mass function of a Poisson distribution in the second line and used the abbreviation $\alpha = \exp(-w^T F^{(i)}\Delta t)$. This is simplified to

$$\log p(Y|F,w) = \sum_i \left[-Y^{(i)}w^T F^{(i)}\Delta t - \exp(-w^T F^{(i)}\Delta t) - \log\left(Y^{(i)}!\right)\right] \ .$$

Note that in the main text (Equation 11), for simplicity we absorbed $-\Delta t$ into the weights, i.e.

$$w' := -\Delta t w \, .$$

5 Event probabilities and rates

Given a transition rate $\lambda(t, F(t))$ which depends on other variables $F(t)$, what is the probability (referred to as $P(t)$ in the main text) of a state transition happening in the interval $[t, t+\tau]$?

The number $N_t^{t+\tau}$ of transition events happening in a single in $[t, t+\tau]$ obeys an inhomogeneous Poisson process with time-dependent rate $\lambda(t, F(t))$, i.e. it has probability mass function

$$P\left(N_t^{t+\tau} = k\right) = \frac{m(t,\tau)^k}{k!} e^{-m(t,\tau)} \ ,$$

where

$$m(t,\tau) = \int_t^{t+\tau} \lambda(s, F(s))ds$$

is the rate integrated over the period of interest. We are interested in $k = 0$ (no event happens in $[t, t+\tau]$)

$$P\left(N_t^{t+\tau} = 0\right) = e^{-m(t,\tau)} \ .$$

From that it follows that the probability (termed $P(t)$ in the main text) of one or more events happening in $[t, t+\tau]$ is

$$P\left(N_t^{t+\tau} > 0\right) = 1 - e^{-m(t,\tau)} \ .$$

Note that for our model, only one state transition can occur (i.e. if the cell changed its state from I to II, it cannot undergo the transition ever again) but the above formula remains valid, since the probability of more than one event happening in $[t, t+\tau]$ is of order $m^2(t,\tau)$ and hence neglegible for small τ.

References

[1] Dodd, P. J. and Ferguson, N. M. (2009). A many-body field theory approach to stochastic models in population biology. *PloS one*, **4**(9), e6855.

[2] Jeffreys, H. (1939). *The Theory of Probability*. Oxford University Press.

[3] Kendall, M. G. and Stuart, A. (1967). *The advanced theory of statistics*. Number Bd. 1 in The Advanced Theory of Statistics. Griffin.

[4] Murphy, K. P. (2012). *Machine learning: a probabilistic perspective*. The MIT Press, Cambridge, Massachusetts.

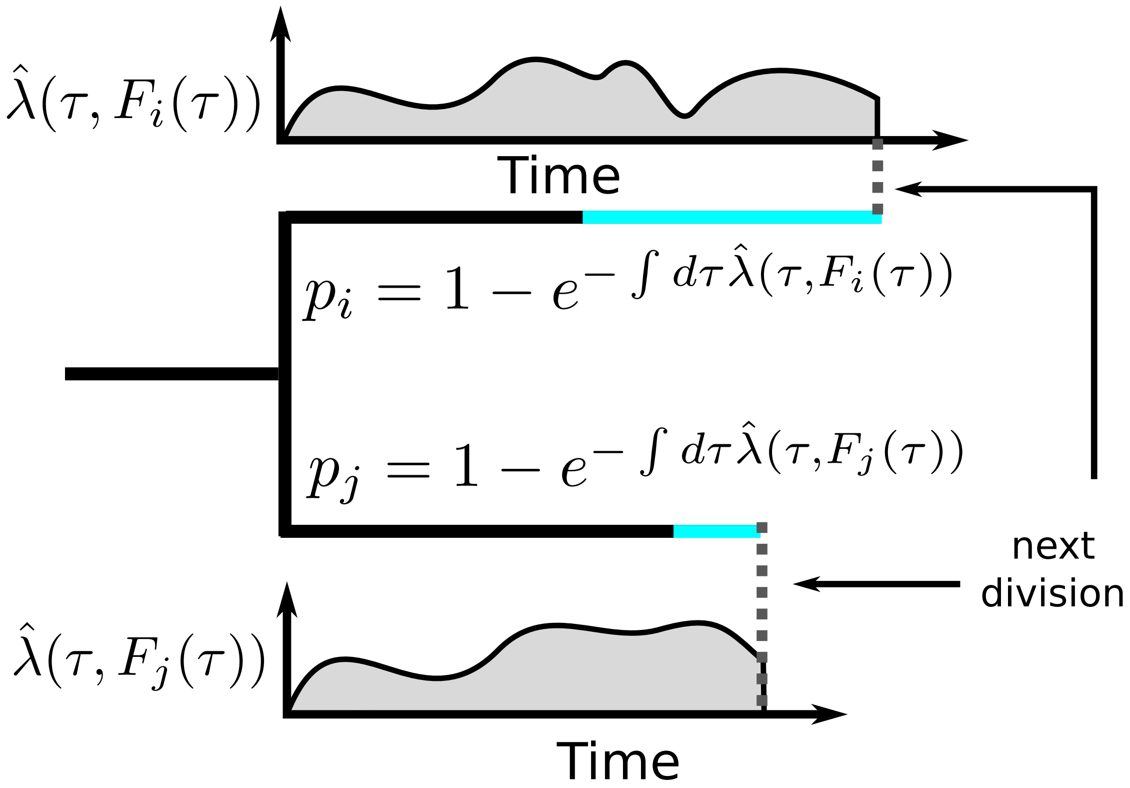

Figure 1: Calculating the expected frequencies of sister subtrees patterns. For a pair of sister cells i, j, the individual transition rates $\hat{\lambda}_i, \hat{\lambda}_j$ of both cells are evaluated across their cell cycle using the previously estimated parameters and yield the probabilities p_i, p_j of each cell to undergo a state transition.

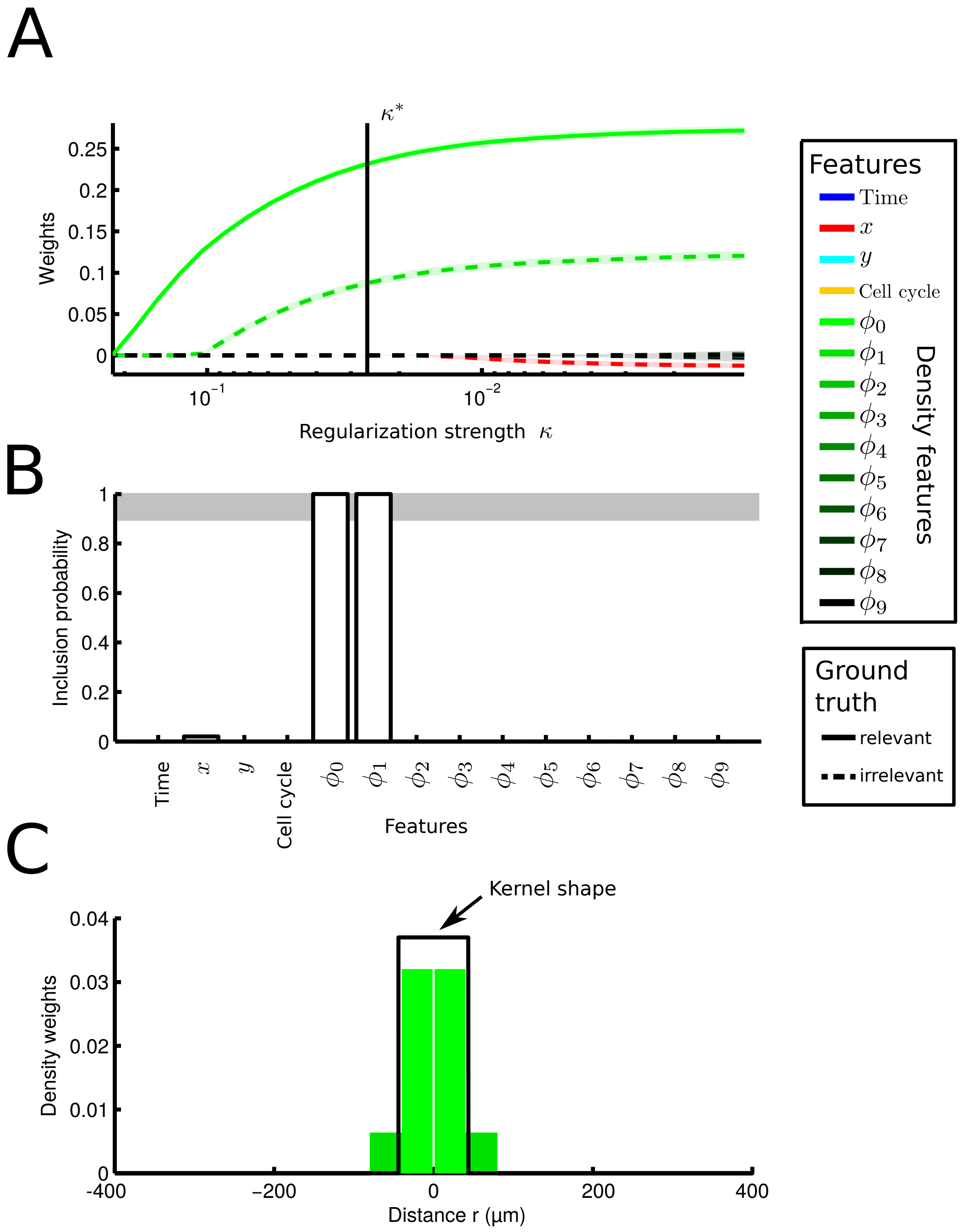

Figure 2: Regularized generalized linear models (GLMs) correctly identify a short range spatial interaction. A) Regularization path of the GLM applied to a density dependent dataset with a short range tophat-kernel ($R = 40\mu m$). The optimal regularization strength κ^* determined by cross validation is shown as a vertical black line. Solid (dashed) lines correspond to relevant (irrelevant) features in the respective scenario. B) Inclusion probabilities of the features determined from 50 bootstraps. The model correctly identifies ϕ_0 while ϕ_1 is wrongly included into the mode. C) Inspecting the reconstructed kernel of local cell density (bars) reveals that the shape of the kernel is reasonably approximated and the contribution of the irrelevant feature ϕ_1 to the kernel is small.

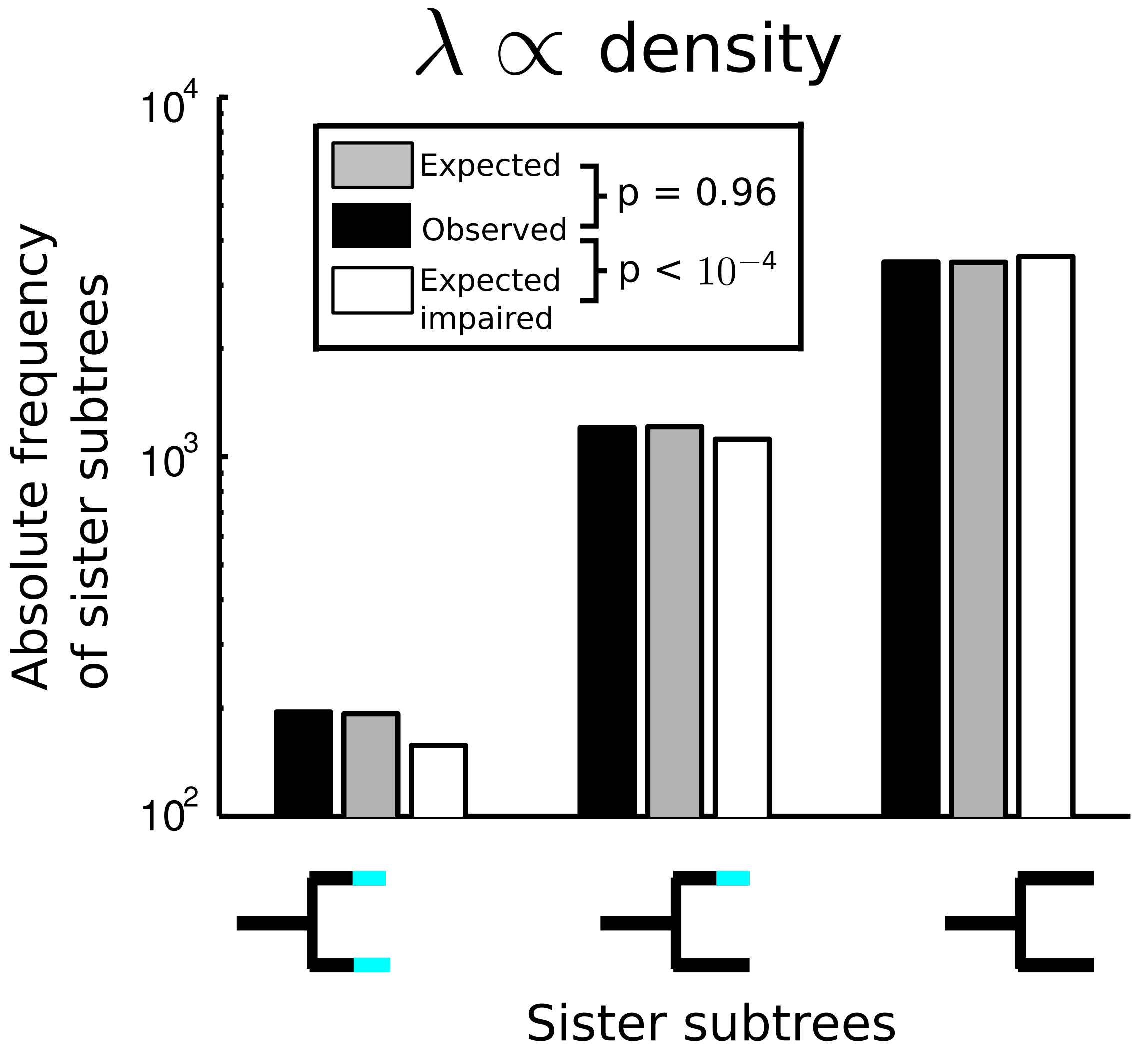

Figure 3: Observed and expected frequencies of sister pairs for the density dependent scenario and a reduced sample size of 2000 onsets. Compare to Figure 7A of the main text, which shows the analogous plot for a sample size of 4500 onsets.

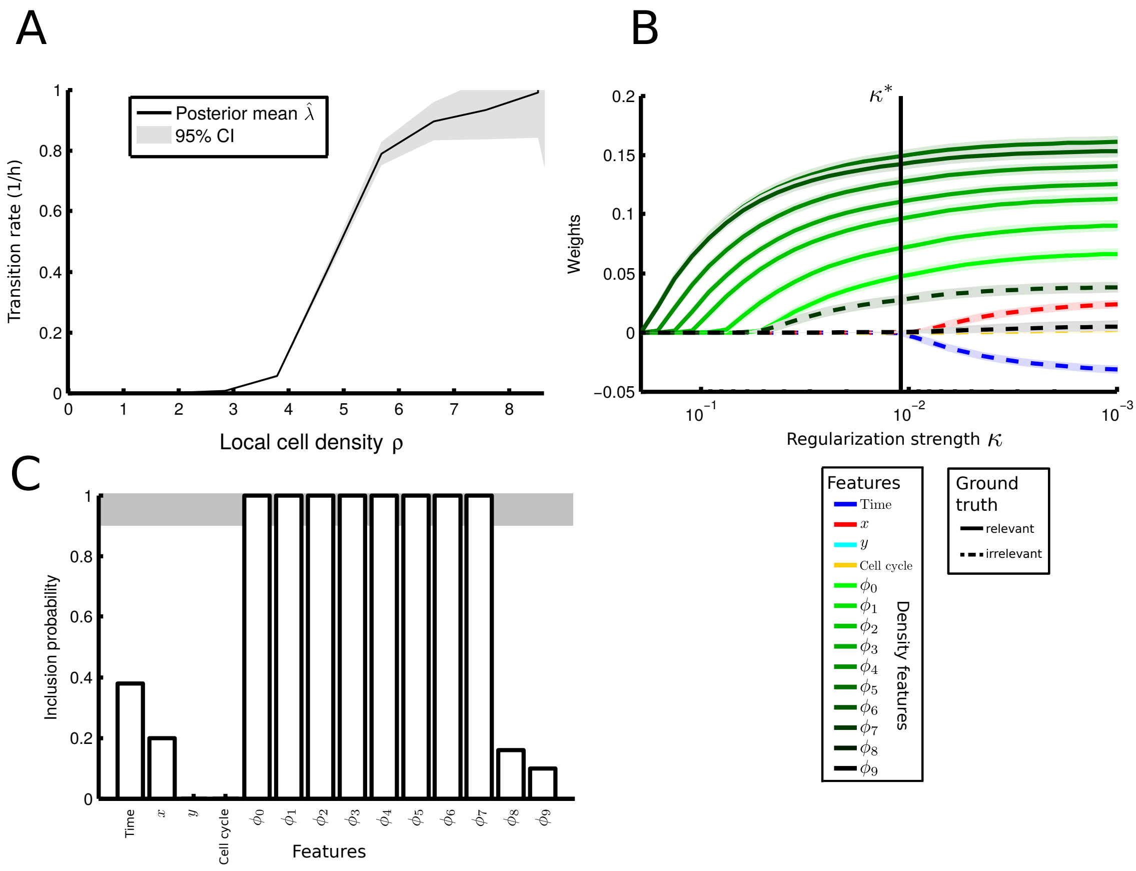

Figure 4: The model performance for a nonlinear (sigmoid) transition rate $\lambda(\rho) = \frac{1}{1+(K_D/\rho)^n}$ with $K_D = 5$ and $n = 5$. A) The transition rate as a function of cell density ρ estimated non-parametrically using Eq. (10) of the main text. B) The regularization path of the GLM fitted to this dataset (see also Figure 4 of the main text). C) The inclusion probabilities for each feature show that despite the strongly nonlinear transition rate, the method can identify all relevant density features and only includes one irrelevant feature (ϕ_7) into the model.
